# Atomistic Simulation Informs Interface Engineering of Nanoscale LiCoO_2_

**DOI:** 10.1021/acs.chemmater.2c01246

**Published:** 2022-08-19

**Authors:** Spencer Dahl, Toshihiro Aoki, Amitava Banerjee, Blas Pedro Uberuaga, Ricardo H. R. Castro

**Affiliations:** † Department of Materials Science and Engineering, University of California, Davis, California 95616, United States; ‡ Irvine Materials Research Institute (IMRI), University of California, Irvine, California 92697, United States; § Department of Metallurgical and Materials Engineering, Indian Institute of Technology, Jodhpur, Rajasthan 342030, India; ∥ Materials Science and Technology Division, Los Alamos National Laboratory, Los Alamos, New Mexico 87545, United States

## Abstract

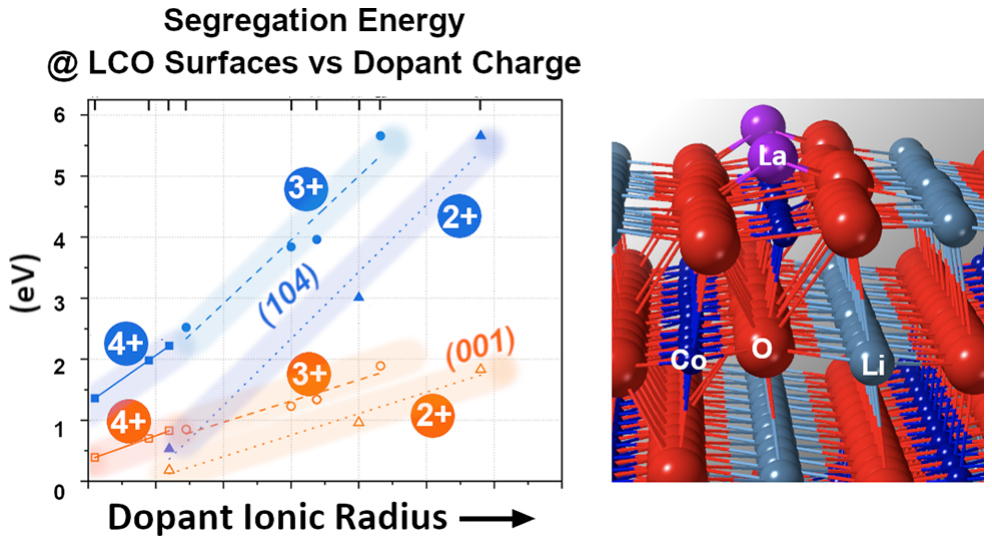

Lithium-ion batteries continue to be a critical part of the search for enhanced energy storage solutions. Understanding the stability of interfaces (surfaces and grain boundaries) is one of the most crucial aspects of cathode design to improve the capacity and cyclability of batteries. Interfacial engineering through chemical modification offers the opportunity to create metastable states in the cathodes to inhibit common degradation mechanisms. Here, we demonstrate how atomistic simulations can effectively evaluate dopant interfacial segregation trends and be an effective predictive tool for cathode design despite the intrinsic approximations. We computationally studied two surfaces, {001} and {104}, and grain boundaries, Σ3 and Σ5, of LiCoO_2_ to investigate the segregation potential and stabilization effect of dopants. Isovalent and aliovalent dopants (Mg^2+^, Ca^2+^, Sr^2+^, Sc^3+^, Y^3+^, Gd^3+^, La^3+^, Al^3+^, Ti^4+^, Sn^4+^, Zr^4+^, V^5+^) were studied by replacing the Co^3+^ sites in all four of the constructed interfaces. The segregation energies of the dopants increased with the ionic radius of the dopant. They exhibited a linear dependence on the ionic size for divalent, trivalent, and quadrivalent dopants for surfaces and grain boundaries. The magnitude of the segregation potential also depended on the surface chemistry and grain boundary structure, showing higher segregation energies for the Σ5 grain boundary compared with the lower energy Σ3 boundary and higher for the {104} surface compared to the {001}. Lanthanum-doped nanoparticles were synthesized and imaged with scanning transmission electron microscopy-electron energy loss spectroscopy (STEM-EELS) to validate the computational results, revealing the predicted lanthanum enrichment at grain boundaries and both the {001} and the {104} surfaces.

## Introduction

Lithium-ion batteries continue to be an integral part of the rechargeable battery industry and the search for sustainable energy storage. Although lithium-ion technologies have been widely utilized over the past few decades, energy content and charging rates are still insufficient to meet automotive energy demands.^1^ Nanomaterials offer potential improvements to enhanced battery operation kinetics through the increased surface area, shortening of diffusion path lengths, and increased rates of lithium intercalation.^2^ However, the main degradation mechanisms, transition metal dissolution, reactivity to the electrolyte, and intergranular cracking, are exacerbated at the nanoscale, leading to catastrophic decreases in capacity after a few cycles.^3^

Many of the problems in nanoscale cathodes directly result from their thermodynamic instabilities. A significant fraction of atoms are located at interfacial regions in nanomaterials, bringing intrinsic excess energies to the system.^4,5^ A potential method for stabilizing surfaces and grain boundaries is the compositional design to provoke dopant segregation, also known as interfacial excess. Following derivations from the Gibbs adsorption isotherm,^6^ interfacial excesses of solid solutes can reduce stress energies and increase the overall stability of nanomaterials.^7^ Nakajima et al. recently explored scandium doping of LiMn_2_O_4_ nanoparticles and directly measured the doping effects on surface and grain boundary energies.^8^ The data showed decreasing interfacial energies with the scandium doping and preferential scandium segregation to the grain boundaries. The results align with other studies using this ‘interfacial engineering’ to stabilize catalytic supports and other nanostructured oxides.^9,10^ In parallel, Wang et al. showed that dopant segregation enhances cathodes’ cyclability through suppressed intragranular cracking and increased mechanical strength.^11^ Although the authors did not discuss interfacial energies, interfacial segregation always has a cause–effect relationship with the local energies. The work exploits the relationship between interfacial mechanical strength and thermodynamics, as recently reported.^12,13^ It is important to note that interfacial excess differs from coating technologies.^14^ The first is a spontaneous phenomenon driven by thermodynamics that does not require additional processing steps and does not constitute a separate phase.

There is still an overall lack of thermodynamic data on dopant segregation correlations with interfacial energies in relevant technological systems, such as lithium-ion structures, to enable effective design for performance.^15−17^ In this work, we used atomistic simulations to study relevant interfaces in nanoscale LiCoO_2_ (LCO) to investigate the segregation potentials of dopants to surfaces and grain boundaries. The goal is to inform experiments regarding dopant selection criteria for interfacial energy design. Two representative surfaces, {001} and {104}, and two low-index grain boundaries, Σ3 and Σ5, were constructed using atomistic models and energetically minimized. Different dopants substituted individual cobalt sites in the structure to map the simulation cell energy at different dopant positions. Divalent, trivalent, and tetravalent dopants with different ionic radii were introduced into the systems to explore the physical–chemical impacts on the relative segregation energy. Overall, dopants showed higher segregation energy at {104} surfaces than at {001}, and higher segregation energies for Σ5 as compared to Σ3. Moreover, the segregation energies increased with the atomic radius. Informed by the simulation results, LCO nanoparticles were synthesized and doped with the element with the highest segregation energy, lanthanum. The results suggest simulations can satisfactorily predict segregation in cathode materials despite the assumptions made, but more quantitative segregation experiments are needed to establish more reliable models for engineering applications.

## Methods

### Atomic Simulations

The atomistic simulations were performed within the LAMMPS framework,^18^ and all simulations were conducted with three-dimensional periodic boundary conditions in all directions. We applied standard Coulomb–Buckingham potentials to model the two-body atomic interactions.^19^ The Buckingham potential models the energy for the short-range interactions between particles. The additional Coulombic potential term models the electrostatic potential energy of the long-range interaction between ionic charges summed using Ewald’s method.^20^ The cutoff distance for all two-body interactions in the simulations was 8.0 Å, and the Buckingham potential parameters for all species considered are shown in Table 1. We note that while there are other potentials for the Li–Co–O system, including some that describe charge transfer,^21,22^ this parameter set is the only parameterization we found for which LCO was stable and that had transferable parameter sets consistent with the same O^2–^–O^2–^ interaction for the dopant species.

**Table 1:** Interatomic Pair Potential Parameters for LCO and Dopant–Oxygen Interactions in the Buckingham Coulomb Potential

ionic pair	*A* (eV)	ρ (Å)	*C* (eV × Å^6^)
O^2–**...**^O^2–^^23^	22764.3	0.149	43.0
Li^+**...**^O^2–^^23^	15785	0.1964	0
Co^3+**...**^O^2–^^23^	1195	0.3087	0
La^3+**...**^O^2–^^24^	1545.21	0.3590	0
Gd^3+**...**^O^2–^^25^	1885.75	0.3399	20.34
Y^3+**...**^O^2–^^26^	1310.00	0.3561	0
Sc^3+**...**^O^2–^^27^	1337.63	0.34303	0
Ti^4+**...**^O^2–^^28^	754.2	0.3879	0
Sn^4+**...**^O^2–^^29^	938.7	0.3813	0
Zr^4+**...**^O^2–^^30^	1057.03	0.376	0
Mg^2+**...**^O^2–^^31^	821.60	0.3242	0
Ca^2+**...**^O^2–^^31^	1228.90	0.3372	0
Sr^2+**...**^O^2–^^31^	1400.0	0.3500	0

**Figure 1:**
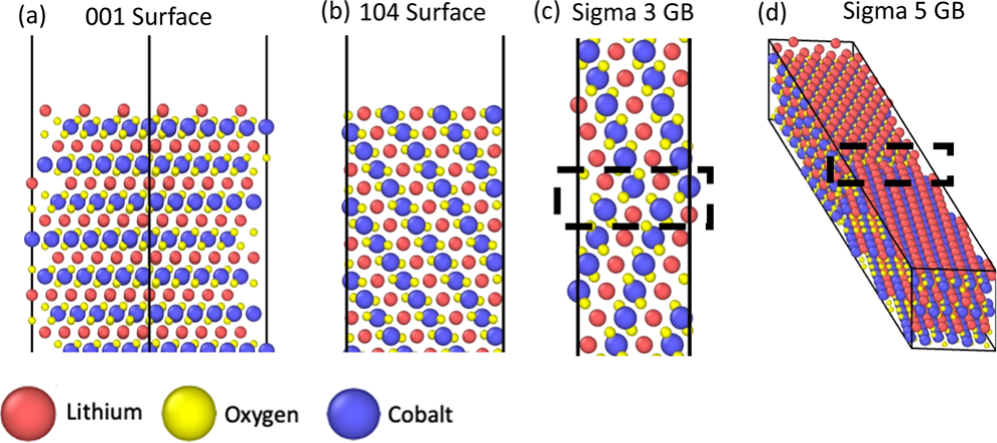
Structures of the LCO interfaces used for the atomic calculations. (a) {001} Surface, (b) {104} surface, (c) Σ3 grain boundary, and (d) Σ5 grain boundary. The dashed boxes denote the coincidence site lattice of the grain boundary between the two grains.

The layered O3 trigonal LiCoO_2_ (Space Group *R*3̅*m*) unit cell was obtained from The Materials Project (ID: mp-22526).^32^ Two low-index surfaces and grain boundaries were constructed to study the segregation profile of ten different dopants. The two design constraints used for building the interfaces were (a) maintaining the stoichiometry of the structure by not deleting or adding any atoms and (b) modifying polar surfaces to remove any surface dipoles. One polar surface, {001}, and one nonpolar surface, {104}, were studied due to their stability, prevalence in the LCO structure, and expected low surface energies.^33^ For the polar {001} surface, several terminations could be considered based on the cleavage plane chosen. According to Hu et al., the cobalt layer termination is an unstable configuration that causes a mix of trivalent and tetravalent cobalt ions on the surface layer, leading to numerous surface configurations of cobalt ions with different oxidation states.^34^ The two possible oxygen terminations also have low stability and require a strongly reducing environment to stabilize the surface oxygen. Due to the instability of the cobalt and oxygen terminations, the lithium termination is the preferred orientation for the {001} surface.^35^ One crucial consideration of the slab geometry for Tasker Type III surfaces, such as the {001} surface studied here, is to prevent surface dipole moments that cause the surface energy to diverge.^36^ The surface dipole is counteracted by moving half of a monolayer of lithium from the top surface to the bottom surface; the resulting surface is illustrated in Figure 1a. As described by Kramer and Ceder,^35^ that structure has an equal charge of +1/2 at both surface layers and a net charge of −1 in the bulk. This leads to a global charge balance of the stoichiometric slab while ensuring Co remains in the trivalent oxidation state. It also provides that the two resulting surfaces have a very similar, if not identical, atomic structure. The vacancy configuration of the surface was modeled after the work of Ceder and Van der Ven and moved every other lithium row to the opposite surface of the structure.^37^ This configuration of the surface lithium atoms is the lowest surface energy arrangement that Ceder and Van der Ven constructed. The designed slab had dimensions of 1.7 × 1.5 × 5.5 nm^3^ with 0.85 nm of skew in the *xy* plane and 2 nm of vacuum introduced for both the top and bottom surfaces.

For the nonpolar Tasker Type I {104} surface,^36^ there is only one possible termination of O–Li–O–Co and no surface dipole to cause surface energy divergence. The structure dimensions for this surface were 1.7 × 1.7 × 4.6 nm^3^ with 2 nm of vacuum introduced at both surfaces, as shown in Figure 1b. The same LCO (Space Group *R*3̅*m*) structure from Materials Project was used to create the slab surface model of the {104} surface.

Two low-index grain boundaries were also studied to understand dopant segregation profiles and interface stabilization at grain boundaries. An atomic model of a Σ3 grain boundary of LCO was constructed using GB-code^38^ and VESTA,^39^ with a common rotation axis of {110} and an orientation plane of {11̅2} (Figure 1**c**). We considered the conventional cell of LCO first to construct the Σ3 GB using GB-code without specifying the chemical identity of the atoms. Next, we used VESTA to assign the chemical identities. That boundary represents the simplest and lowest energy GB structure in most materials and has dimensions of 0.8 × 1.0 × 10.3 nm^3^.

The Σ5 grain boundary, representing a higher energy interface but still structurally simple, was designed using the Aimsgb Python framework for building periodic grain boundaries.^40^ The tilt boundary was constructed with a common rotation axis between the two grains along the {001} plane and by orienting the grain boundary plane along the {120} plane. An additional interfacial distance of 1.0 Å was added between the two grains to prevent overlapping atoms and allow the minimizations to converge. The structure dimensions were 0.8 × 8.8 × 1.4 nm^3^, with an *xy* skew of 3.2 nm, as shown in Figure 1d.

All four designed structures were energetically minimized by anisotropically relaxing the atoms and simulation cells before any dopant replacements. The grains were translated in both directions parallel to the grain boundary in 0.1 Å increments and energetically minimized at each position for the two grain boundaries. The γ surface mapping provides an energy landscape of the grain boundary with respect to the relative translation of the grains. The lowest energy structure was used for the dopant studies.

The dopants selected for this study covered a range of ionic radii and oxidation states: isovalent dopants were chosen (Sc^3+^, Y^3+^, Gd^3+^, La^3+^), as well as six aliovalent dopants consisting of three divalent dopants (Mg^2+^, Ca^2+^, Sr^2+^) and three tetravalent dopants (Ti^4+^, Sn^4+^, Zr^4+^). The segregation profiles of these dopants were studied by replacing one Co^3+^ atom with a dopant and allowing the structure to relax through energy minimization while holding the simulation cell dimensions constant. The process was repeated, one by one, for each Co^3+^ in the structure, and the system’s energy was computed for each dopant position. The difference between the energy of a dopant in the bulk compared to the dopant at a surface or a grain boundary was used to calculate the segregation energy (*E*_seg_).
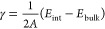
1

The surface energy or grain boundary energy (γ) of the undoped interfaces was calculated by finding the energy difference between a slab with two interfaces (surfaces/grain boundaries, (*E*_int_) and a bulk slab geometry with the same number of atoms (*E*_bulk_)). This is shown in eq 1,^41^ where 2*A* accounts for the interfacial area of the two surfaces/grain boundaries.

### Experimental Section

Doped and undoped nanoparticles of LCO were synthesized by adapting protocols developed by Okubo et al.^3^ The coprecipitation method was performed by dissolving 20 mmol of Co(NO_3_)_2_·6H_2_O into 100 mL of deionized (DI) water and preparing a 100 mL of 5 M NaOH solution. For the doped nanoparticles, the amount of cobalt nitrate was reduced and replaced with 1 or 2 mol % of the dopant in the nitrate form. The nitrate solution was slowly added to the basic NaOH solution to precipitate the Co(OH)_2_ nanoparticles and then diluted into 1,800 mL of DI water. The diluted suspension was oxidized by bubbling air through the stirred suspension for 48 h to yield the CoOOH nanoparticles. The CoOOH nanoparticles were centrifuged and washed with DI water 5 times and dried at 80 °C overnight. The precipitates were ground in a mortar and pestle, and 500 mg was stirred into a 133 mL aqueous solution containing 1 M LiOH. The suspension was added to a 200 mL stainless steel autoclave with a PTFE liner and placed in a furnace. The furnace was heated to 180 °C at 0.5 °C/min and held for 12 h, then the autoclave was cooled at 1 °C/min to 100 °C and removed to cool at room temperature. The LCO precipitate was washed and centrifuged in water 4 times and dried at 80 °C overnight.

X-ray diffraction patterns were obtained with a Bruker AXS D8 Advance powder diffractometer (Cu Kα radiation, λ = 1.5406 Å) at 40 kV and 40 mA. Jade MDI software was used to confirm crystallographic phases and lattice constants. Crystallite sizes were calculated using the Scherrer equation using whole profile fitting.^42^ Raman spectra were collected on a Renishaw confocal Raman microscope with a 785 nm laser at 50% intensity and 30 s measurement time. Scanning transmission electron microscopy (STEM) coupled with electron energy loss spectroscopy (EELS) revealed the morphology of the nanoparticles and mapped dopant distribution. JEOL Grand ARM 300CF equipped with Gatan GIF Quantum with K2-summit was used for the study, operating at 300 keV.

## Results: Atomistic Simulations

**Figure 2:**
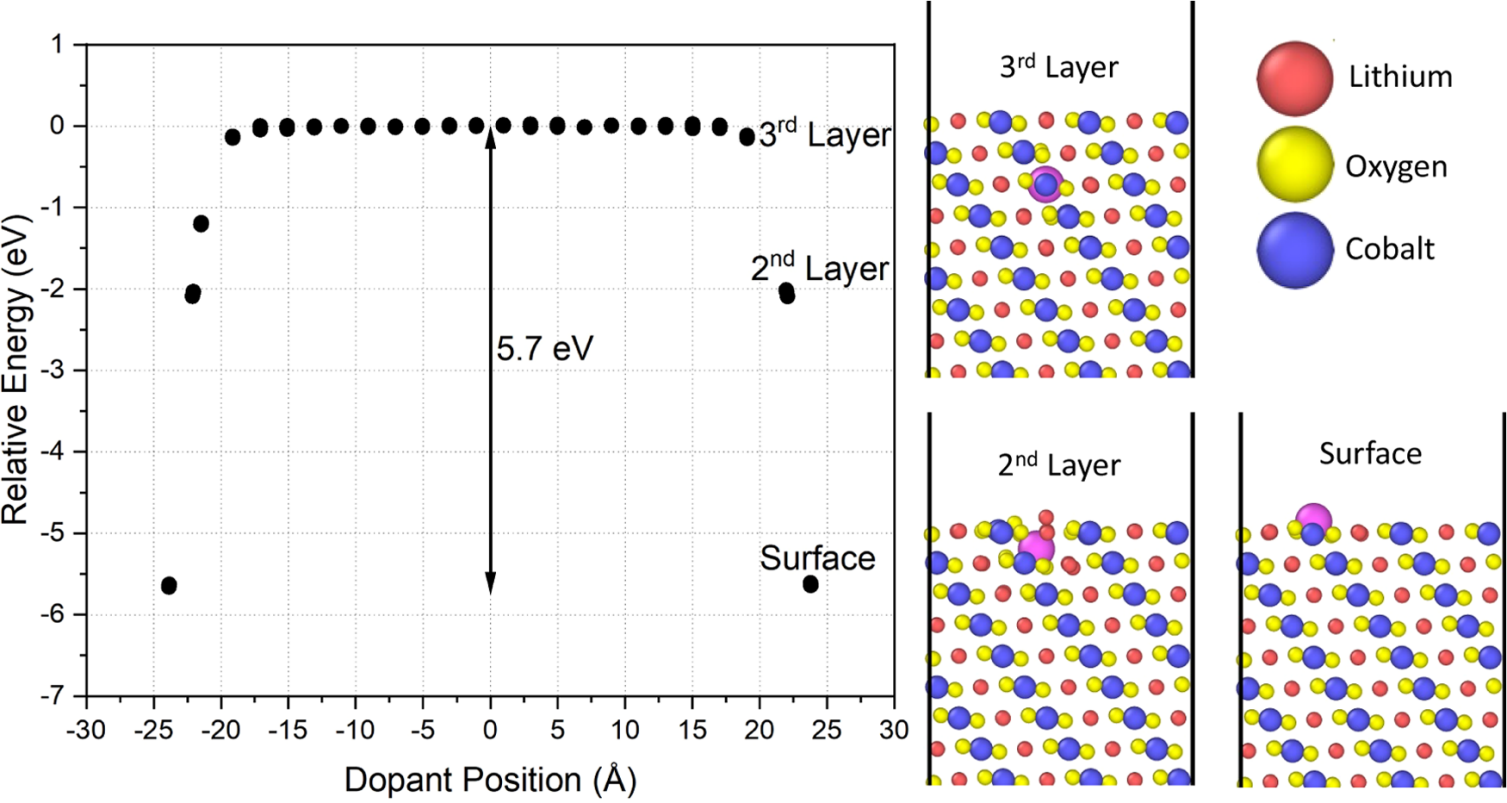
Segregation profile of La^3+^ doping each cobalt position in the {104} surface structure. The structure has two surfaces on either side of the simulation cell. The images depict the dopant position near the top surface at 23.8 Å.

The first studies focused on the segregation potential of isovalent and aliovalent dopants on the minimized LCO surface structures. Figure 2 shows an example segregation profile acquired for La^3+^ at the nonpolar {104} surface. The plot shows the minimized energy of the system versus the dopant position in the crystal structure. Each cobalt atom was substituted by La^3+^ one at a time, and the structural energy was minimized to evaluate the most favorable replacement site. The presented graph had surfaces on both sides of the cell, at +24 and −24 Å, with the positions near 0 Å representing the crystal bulk. In these calculations, the dopant minimizes the system energy further when placed near the surfaces. The energy difference between the state with the dopant replaced in the bulk value and the surface substituted dopants gives the segregation energy for the individual atom, which is 5.7 eV for La^3+^ at the {104} surface. These simulations also explain the energetic trends and associated structural arrangements at and near the surface regions. For example, as seen in Figure 2, La^3+^ ions located at the surface and in the second atomic layer from the surface both protrude outward toward the surface. The behavior shifts the dopant from the cobalt site and can displace other ions around it. The bulk energy values are nearly achieved when La^3+^ is at the third atomic layer from the surface, and the dopant remains close to the initial cobalt position. The relative asymmetry in the plot between surfaces, particularly for the 2nd and 3rd internal atomic layers, refers to local energy minima associated with the large ionic radius of La^3+^. Small shifts in the La^3+^ positions may impact the stability of neighboring sites and, therefore, the system’s overall energy. However, the primary conclusions regarding the most stable sites and the segregation energy are similar for both surfaces.

**Figure 3:**
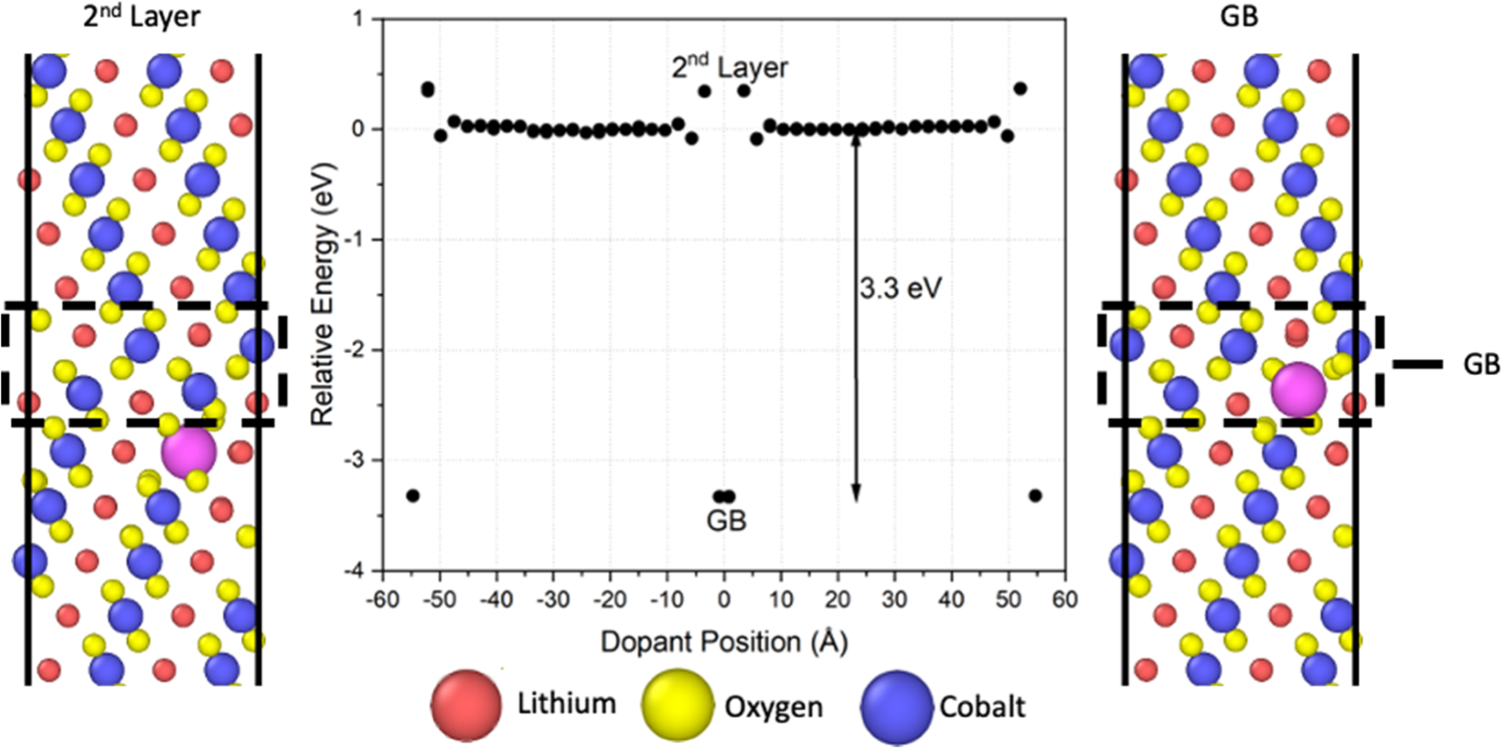
Segregation profile of La^3+^ doping each cobalt position in the Σ3 grain boundary structure. The structure has a grain boundary at the center and a periodic boundary at either end of the simulation cell. Images depict the dopant position at the center boundary and in the layer adjacent to the GB.

Figure 3 shows an example of the energy profile when doping LCO with La^3+^ in the presence of Σ3 grain boundaries. This profile illustrates two grain boundaries, with one in the middle of the structure at 0 Å and another located on the edges of the cell created as a consequence of the periodic boundary conditions. Similar to the surface case, La^3+^ promotes lower energy to the system when segregated to the grain boundary regions. This case results in a spontaneous segregation energy of 3.3 eV, which is slightly lower than the {104} surface and highlights that the dopants may have different affinities for different interfaces based on the thermodynamic stability and coordination of the atoms at the given interface. The calculations also provide insights into the favorable dopant positions. For the Σ3 grain boundary, the system shows the lowest energy when the atoms sit exactly at the interface. However, if substituted in the second atomic layer from the interface, the dopant causes an increase in the energy, suggesting that this substitution is less likely to occur. Since the unfavorable energy is mirrored on both sides of the grain boundary, the phenomenon creates an energetic trap that should limit the dopant mobility across grain boundaries. The pattern was observed for all tested dopants, but the magnitude of the second layer energy deviation depended on their ionic radius. In general, dopants with larger ionic radii, such as lanthanum, presented higher segregation energies (∼3.3 eV) and higher energy aberration in the second layer (∼0.4 eV), while smaller dopants, such as scandium, showed lower segregation energies (∼2.0 eV) and lower energy aberrations (∼0.3 eV).

**Figure 4:**
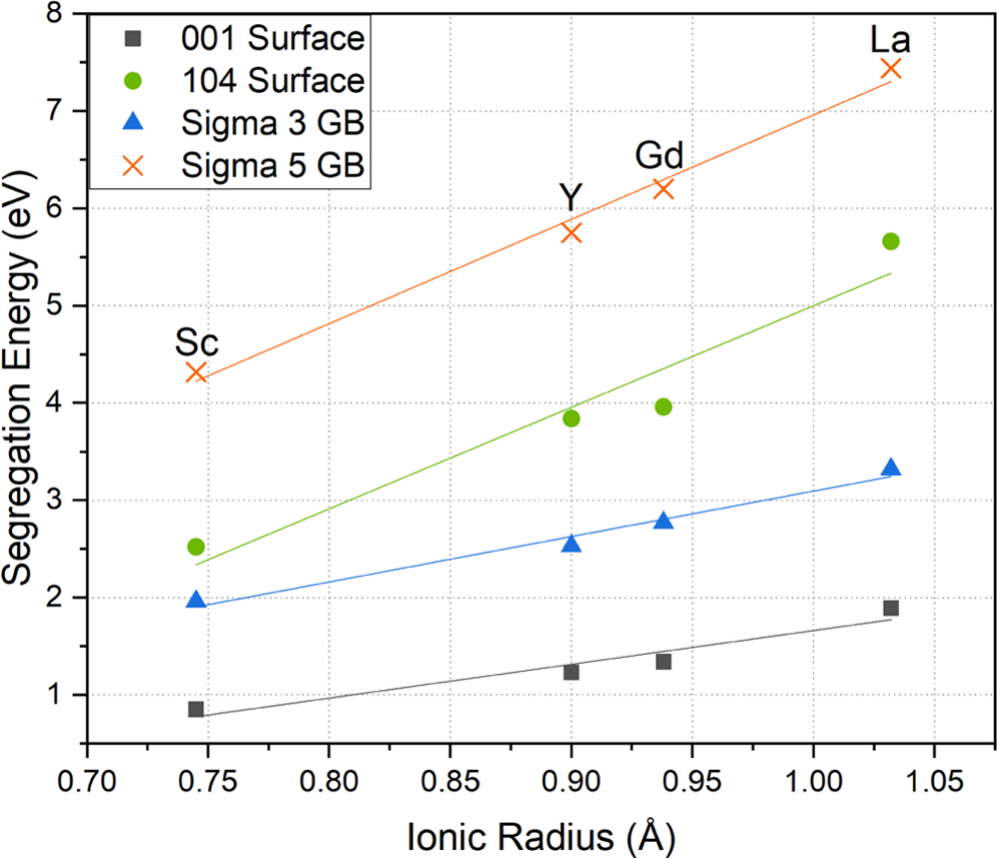
Calculated segregation energies of trivalent dopants (Sc^3+^, Y^3+^, Gd^3+^, La^3+^) plotted against ionic radius for all four constructed interfaces.

Figure 4 shows the compiled results of the segregation energy plotted against the ionic radius of the isovalent dopants for the two surfaces and two grain boundaries. The segregation energy increases with the ionic size of the dopant with a clear, albeit different, linear trend for each of the interfaces in the tested range of ionic radii. The linear behavior likely relates to the elastic strain induced by the dopants when in solid solution and the respective ability of the interfaces to accommodate the dopant at the less coordinated and more disordered placement. The ability of an interface to accommodate a foreign ion is related to its intrinsic thermodynamic stability. According to density functional theory (DFT) studies by Kramer and Ceder, the {001} surface is one of the most stable surface planes in the LCO structure, with a theoretical surface energy of 1.00 J/m^2^ for the termination with a one-half monolayer of lithium at the surface.^35^ Their study also points out the {104} surface is one of the most stable nonpolar surfaces because it has minimal coordination loss compared to other nonpolar surfaces. However, it does have a slightly higher surface energy of 1.05 J/m^2^ compared to the polar {001} surface. This difference could be the cause for the stronger thermodynamic driving force for segregation to the {104} surface. This driving force leads to higher segregation energies to {104} surfaces, a consequent more significant reduction in the surface energy, and an overall more thermodynamically favorable accommodation.

In the present study, the calculated surface energies from eq 1 were 2.21 J/m^2^ for {001} and 1.75 J/m^2^ for {104} surfaces. Despite the numerical differences when compared to Kramer and Ceder’s report^35^ and other first-principles DFT studies,^43^ we also found the surface energies to be close in relative values, with the {001} surface having higher energy. The fact DFT yields lower energies indicates a limitation of the used potentials in the present work. However, those were the only set of potentials that both predicted a stable LCO surface structure and had interactions for the numerous dopant species considered in this study. The relative consistency with recent results, the self-consistency, and experimental confirmations presented later in this work indicate that although the absolute values may be off, the predicted basic physical trends concerning segregation are reliable.

The two studied grain boundaries, Σ3 and Σ5, also presented energetic differences affecting the segregation trends. At the Σ3, the atoms are more coordinated, and the structure shares more atoms at the coincidence site lattice. Equation 1 enabled the estimation of the difference in grain boundary energy between the two structures using the bulk energy of a slab structure with no interfaces and the same number of atoms. From this calculation, the Σ3 boundary showed an excess energy of 0.59 J/m^2^, while the energy for Σ5 boundary was 3.63 J/m^2^. The Σ5 boundary shows significantly higher energy than the Σ3 and supports the inference that the Σ5 is more disordered and atomically less coordinated. High energies are consistent with the covalent nature of LiCoO_2_. The directional characteristic of covalent bonds increases energies due to the significant bond angle distortions. The higher energy leads to stronger segregation potentials, as dopants can alleviate the local stresses by increasing the coordination.

**Figure 5:**
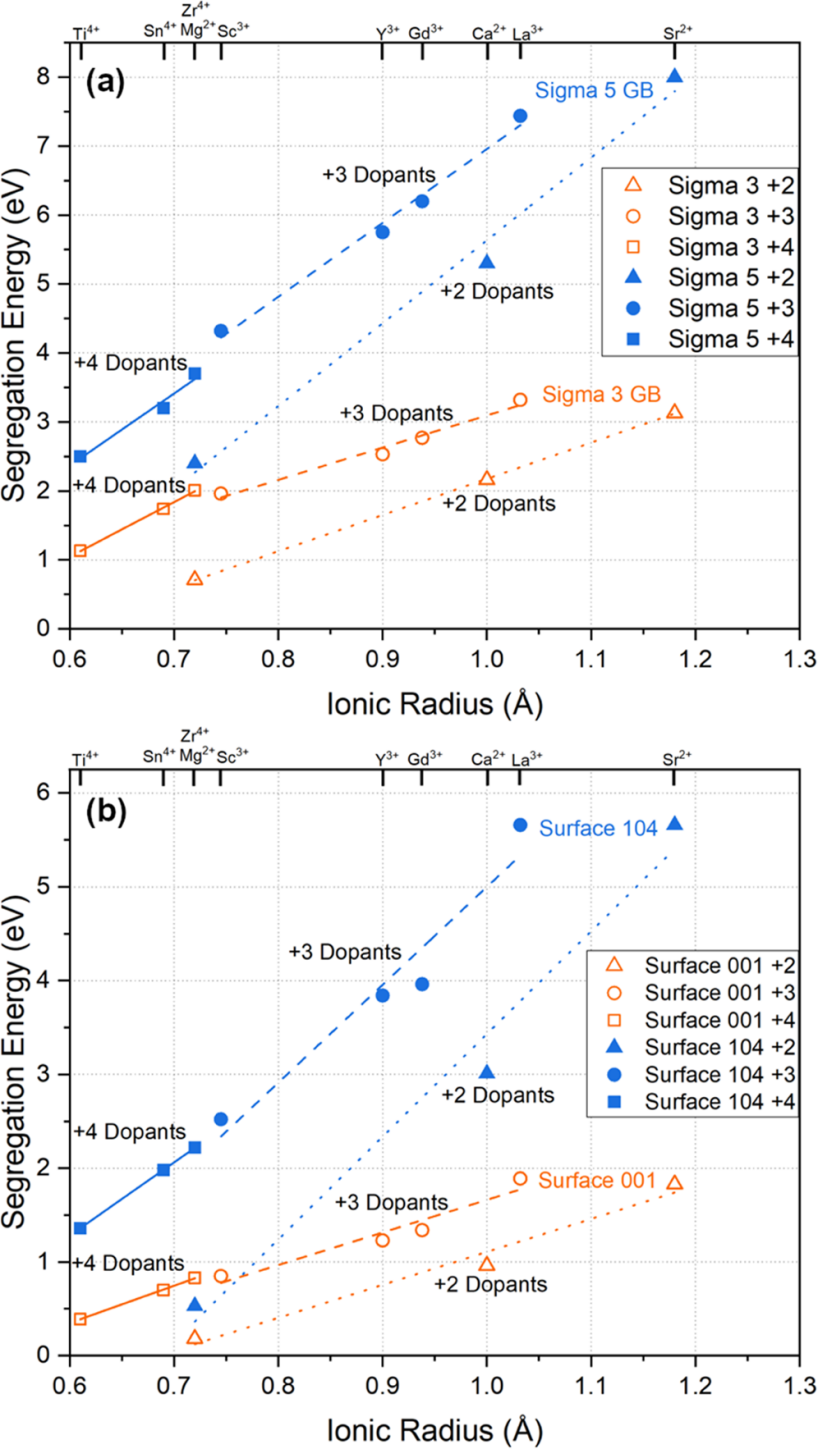
Segregation energies of all divalent, trivalent, and quadrivalent dopants as a function of ionic radius for (a) the two constructed grain boundary structures and (b) the two surface structures.

In addition to the isovalent doping, several aliovalent ions (Mg^2+^, Ca^2+^, Sr^2+^, Ti^4+^, Sn^4+^, Zr^4+^) were tested to study the impact of dopant oxidation state on the segregation behavior. Figure 5a shows the segregation potential of all 10 dopants as a function of the ionic radius for the Σ3 and Σ5 grain boundaries, while Figure 5b shows the segregation potentials for the studied surfaces. A few unique cases from the simulations with aliovalent dopants arose during the dopant replacements, and those are discussed briefly in the Supplemental Information (Figure S1). The linear trend of increasing segregation energy with ionic radius remained consistent for all oxidation states of the dopants. However, the linear dependence is different for each oxidation state and interface, providing interesting insights for dopant selection.

One observation is that the segregation energy increases as the oxidation state of the dopant increases. For example, dopants of similar ionic radius but different charge states, e.g., Mg^2+^ and Zr^4+^, had segregation energies scaling with the charges, i.e., 0.7 and 2.0 eV, respectively, for the Σ3 boundary. Consistently, the Σ5 boundary again had higher segregation energies than the Σ3 boundary due to the higher structural disorder but similar trends with the oxidation state of dopants. Interestingly, results show that all dopants, regardless of the size and charge, had favorable segregation energy. The doped surface structures shown in Figure 5b exhibit similar linear trends to the grain boundaries. This implies all could potentially be used to control interfacial energies, but some had a more pronounced impact.

It is tempting to select the dopants with the highest computed segregation energies, La^3+^ or Sr^2+^, to attempt an interfacial engineering protocol as those would present the highest thermodynamic driving force. However, one should keep in mind that the presented atomistic simulations do not consider the possibility of nucleation of a second phase. As discussed in more detail by Castro,^44^ a saturation of interfacial sites by a dopant can eventually lead to the formation of second phases. The formation of a precipitate is typically undesirable as it compromises electrochemical properties. This was recently observed in La^3+-^doped MgAl_2_O_4_, in which a lanthanum-rich precipitate formed after saturation of the interfacial sites.^45^

While the extremes of segregation energies may not be positive, similarly, low segregation energies, as found for Ti^4+^, which is much closer to the ionic radius of Co^3+^, may not have a high enough segregation potential at dilute concentrations and will provide very little stability enhancement at the interfaces. Additionally, Al^3+^ and V^5+^ dopants were studied due to their small ionic size and ability to enhance some aspects of battery stability (Table S1 and Figure S2).^46,47^ The aluminum dopant shows low segregation energies for both the {104} surface and Σ3 boundary, which follows what has been observed in the literature.^46^ The aluminum dopant has no electrostatic charge or elastic strain to drive the dopant to the interface and therefore remains a bulk dopant. The vanadium not only remains a bulk dopant for the {104} surface but also appears to be thermodynamically unstable at the surface. This could be due to the limitation of a different O^2–^–O^2–^ potential parameter or a strong repulsion on the surface from the higher oxidation state ion. There is a small segregation energy of 0.86 eV for the Σ3 boundary that shows the grain boundaries’ ability to accommodate the excess charge from the vanadium ion. There are mixed results on the ability of vanadium doping to improve electrochemical performance; however, the impact of vanadium as an interfacial dopant in nanoscale materials could be vastly different from bulk doping cathodes.^48^ The segregation energies also show that ions of a similar ionic size to cobalt can still segregate due to the higher oxidation state of the dopant, but the driving force may be small depending on the oxidation state.

## Results: Experiments

**Figure 6:**
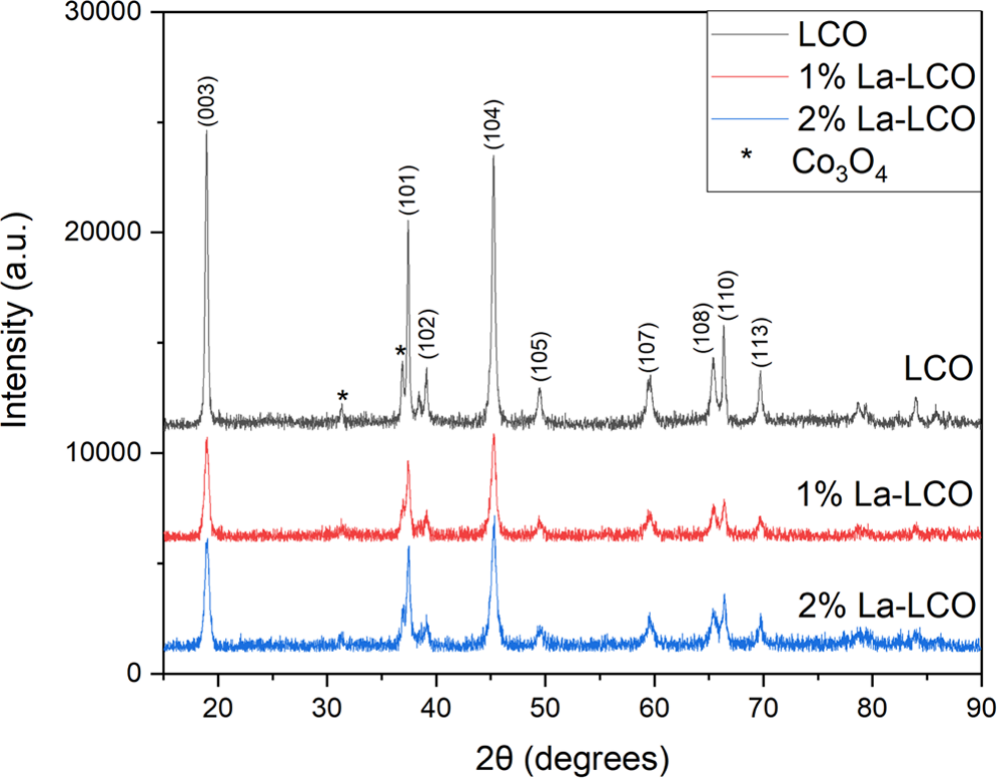
X-ray diffraction patterns of 600 °C calcined undoped LCO, 1, and 2 mol % lanthanum-doped LCO.

**Figure 7:**
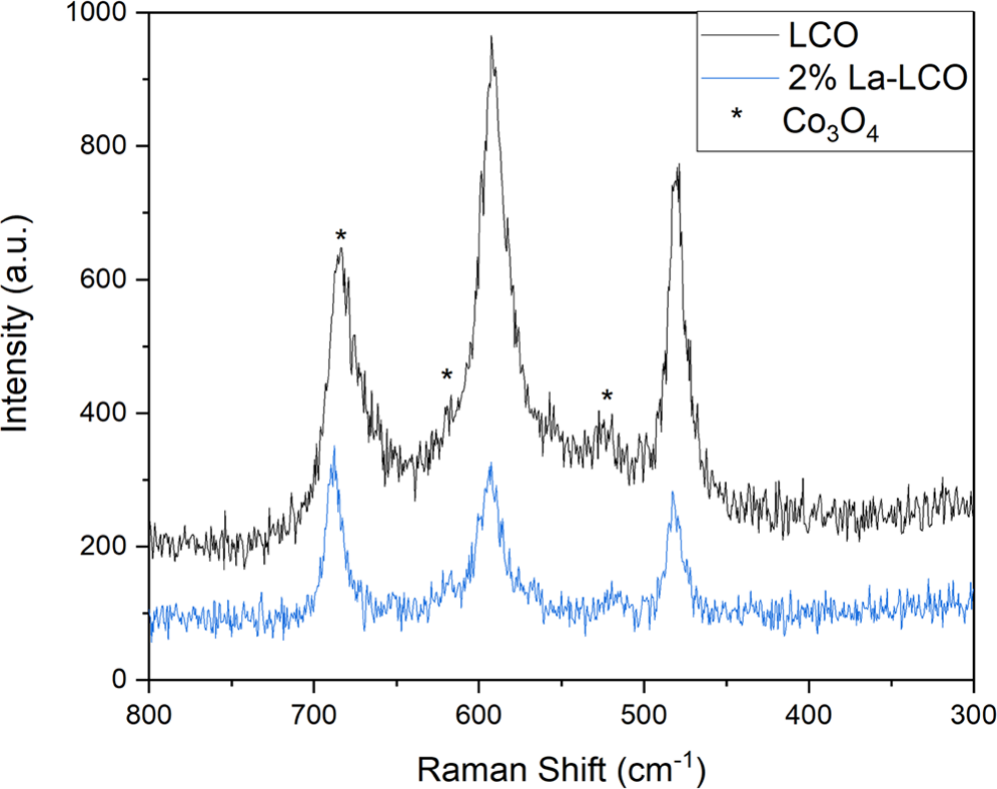
Raman spectra of LiCoO_2_ calcined at 300 °C after synthesis, and 2 mol % lanthanum-doped LiCoO_2_ calcined at 600 °C.

To confirm the segregation predictions, we selected La^3+^ as a dopant at a concentration low enough not to saturate the available interfacial areas, assuming the limit as a monolayer coverage (below 2 mol %). Nanoparticles of lanthanum-doped and undoped LCO were synthesized through a hydrothermal synthesis method. The X-ray diffraction (XRD) patterns of the undoped and doped LCO are shown in Figure 6 and show no evidence of secondary phase formation due to the dopant. Traces of Co_3_O_4_ secondary phase are present in all three samples, but compared to the intensity of the LCO peaks, the amount of the second phase is estimated to be below 1 wt % by Rietveld refinement.^49^ Raman spectra of the doped and undoped LCO in Figure 7 also provide support for no secondary phases caused by excess dopant segregation. The spectra confirm the presence of LCO with the characteristic peaks around 485 and 495 cm^–1^.^50^ The Co_3_O_4_ secondary phase peaks were also confirmed in both samples.^51^ The Raman measurements corroborate the XRD results and show none of the expected lanthanum secondary phases (La_2_O_3_ and LaCoO_3_) forming from excess dopant segregation.^52,53^Table 2 shows the calculated lattice parameters from a whole pattern fitting. The synthesized LCO can crystallize into either a layered or spinel-type structure with similar XRD patterns.^54^ Gummow and Thackeray showed that a *c*/*a* parameter of ∼5.0 indicates a layered type structure, and values closer to 4.9 indicate a spinel-type form. The doped and undoped *c*/*a* parameters are close to 5.0 and show that the doped system maintains the layered structure. The lanthanum doping caused a minimal effect on the parameter *a* and a slight decrease on the parameter *c*. In truth, dopants forming a solid solution within the LCO structure would cause lattice expansion, as observed by Wang et al. when doping with Mn^3+^ or Ni^2+^.^55^ That would be particularly expected in this case since La^3+^ has a significantly larger ionic radius than Co^3+^. Therefore, the lack of structural expansion is already indirect evidence of segregation.

**Table 2:** Calculated Lattice Parameters, Peak Ratios, and Crystallite Sizes from X-ray Diffraction and BET Surface Area for La-Doped and Undoped LCO Calcined at 600 °C

	LCO	1% La-LCO	2% La-LCO
*a* (Å)	2.8174	2.8175	2.8177
*c* (Å)	14.0702	14.0664	14.0656
*c*/*a*	4.994	4.992	4.992
{104}/{003}	0.90	1.05	1.18
crystallite size (nm)	28.1	18.1	17.8
BET surface area (m^2^/g)	20.3	29.1	25.6

The lattice shrinkage could be attributed to the stress induced by the segregated dopants or the observed reduction in crystallite size, as seen in Table 2. The interfacial energy reduction caused by segregation inhibits coarsening driving force independent of the growth mechanisms, leading to smaller crystallite and particle sizes at a given annealing temperature.^56,57^ The results are consistent with the BET surface area shown in Table 2, indicating higher surface areas for the doped samples due to surface stabilization.

The XRD patterns also show La^3+^ doping changes in the relative intensities of certain planes in the LCO structure. In undoped LCO, the ratio of the {104} peak to the {003} is 0.90, but for the doped samples, it is above 1.05. The observation is consistent with the work from Okubo et al., where they show the {003} peak intensity decreases as particle size decreases due to the nanoplatelet morphology of the particles.^3^

**Figure 8:**
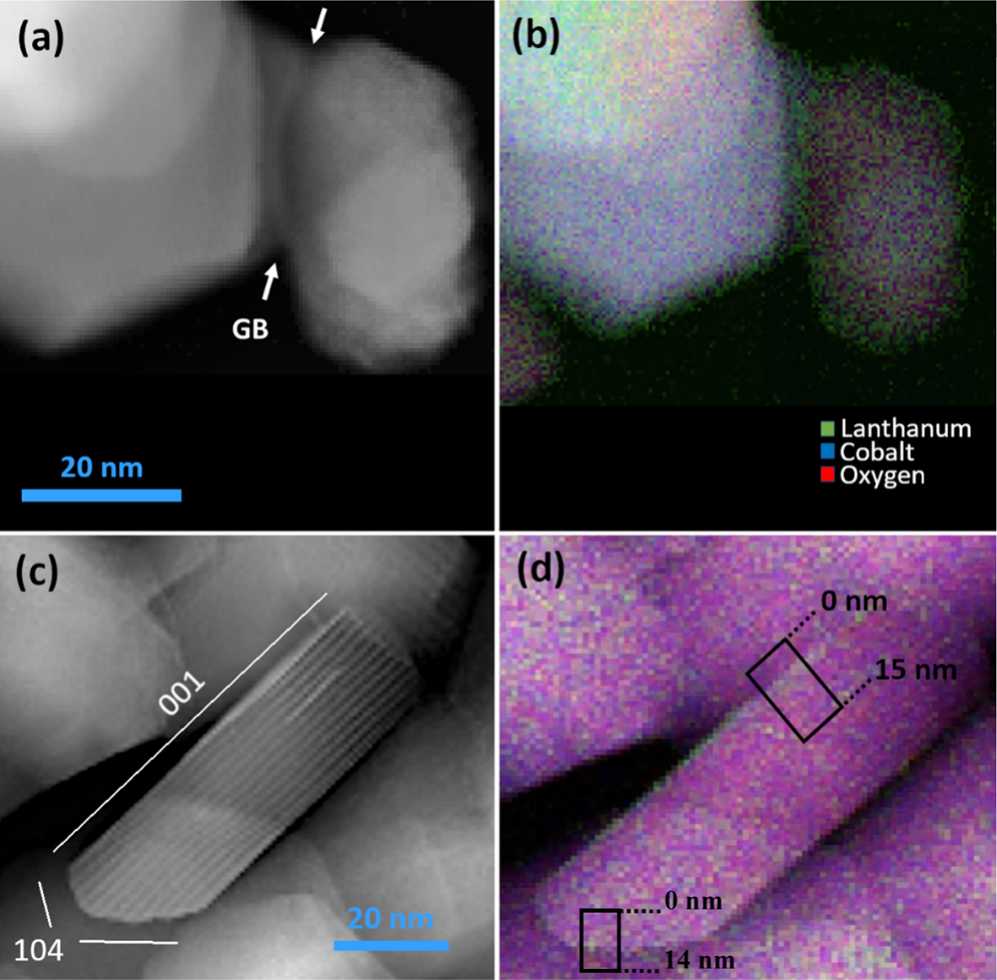
STEM-EELS images of 2% La-doped LCO calcined at 600 °C. Panels (a, c) show the STEM ADF images of the particles, and panels (b, d) show the EELS color mapping of the particles for lanthanum, cobalt, and oxygen. (d) Shows the elemental box scans performed at the interfaces, with the results shown in Figure 9a,b.

Figure 8 shows the STEM and EELS images of the 2% La-doped LCO nanoparticles after calcination at 600 °C. Figure 8a–c confirms the nanoscale dimension and shows the expected nanoplatelet morphology with varying thicknesses of 10–20 nm. Figure 8a indicates that particles are partially connected, with a grain boundary indicated by arrows. Figure 8b shows the EELS composed color mapping demonstrating a concentrated green color around the edges of the particles, depicting the La^3+^ enrichment at both the surfaces and the grain boundaries. The center of the particles had a more purple hue because of the higher fraction of cobalt (blue) and oxygen (red). Figure 8b still shows lanthanum atoms in the center of the nanoparticles. However, most of the nanoparticles in the image are lying flat and showing the {001} surface on the top and bottom of the particle.^43^ The platelike morphology makes it challenging to determine if the lanthanum is at the {001} plane or remains in the bulk structure since electrons are transmitting through the sample. Figure 8c shows a particle oriented perpendicularly, allowing visualization axially along with the *a* parameter to identify the fringes of the *c*-spacing consistently with LCO layered structure. While the top surface is attributed to the {001} plane, the edges of the particles can be assigned to {104} and {012} surfaces.^43^Figure 8**c** also shows evidence of lanthanum enrichment along the {001} surface plane indicated by the phase contrast between cobalt and lanthanum atoms. The segregation of La^3+^ to {001} is confirmed in the color mapping in Figure 8d.

**Figure 9:**
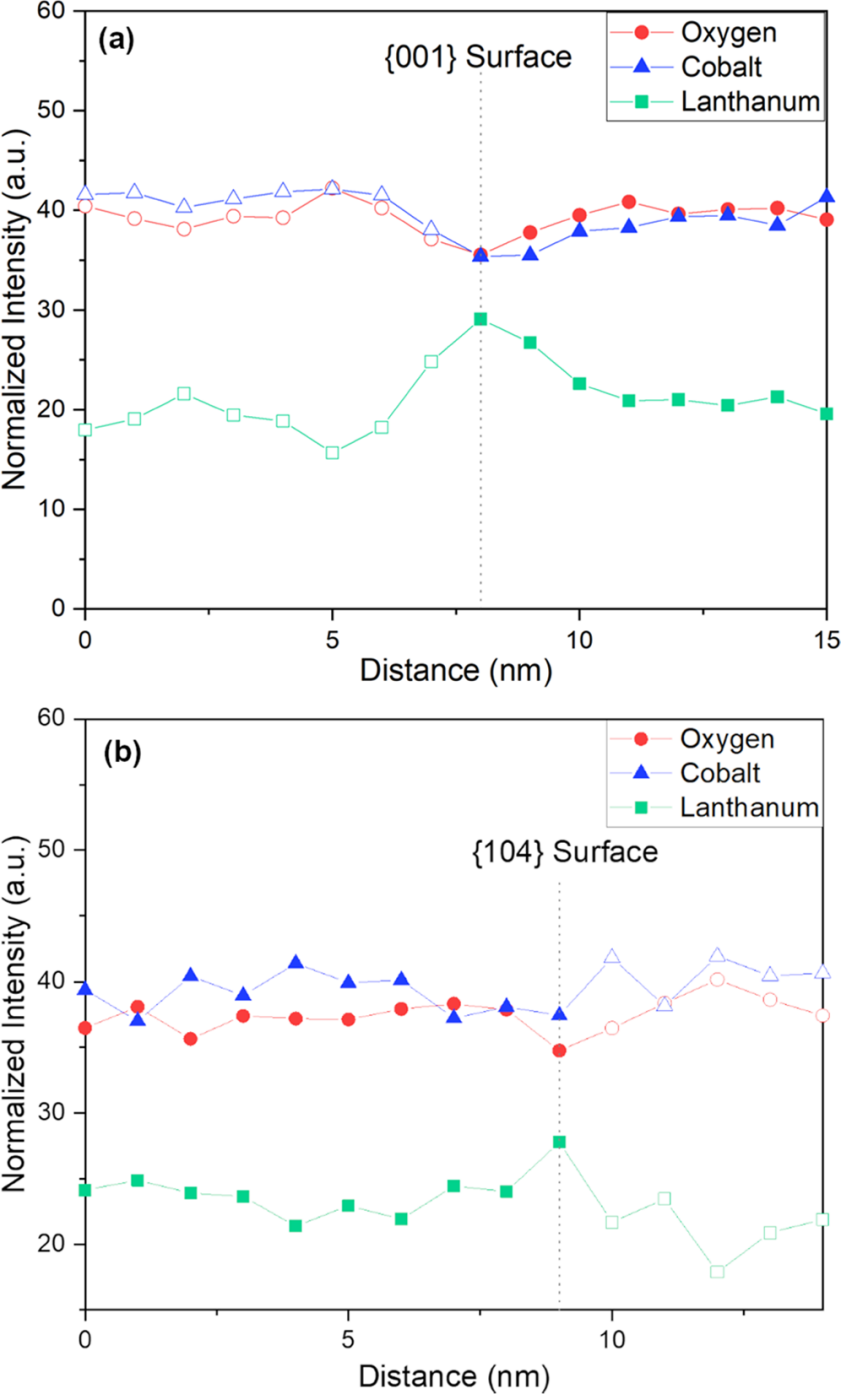
Box scans plotting the atomic concentrations of lanthanum, cobalt, and oxygen of the particles shown in Figure 8d. (a) Box scan of the {001} surface with the dotted line at the 8 nm position portraying the lanthanum enrichment near the {001} surface of the nanoplatelet morphology shown in the figure. (b) Box scan of the {104} surface with the dotted line at 9 nm position portraying the position of the surface. The hollow symbols show the data from the background particles below the platelet being measured, and solid symbols depict the atomic concentrations of the platelet shown in Figure 8d.

Figure 9a shows the box scan measurement of the {001} surface from Figure 8d and displays the highest peak intensity of lanthanum at 8 nm near the surface of the particle. At the same distance, the cobalt and oxygen-normalized intensity dips near the surface, which confirms the lanthanum enrichment near the {001} surface. Note that because the particles overlap (see the box in Figure 8d), the scan shows positive signals for O, Co, and La on either side of the peak position despite the fact the measurement is looking at a surface. Atoms that are from background particles are marked by hollow symbols in the box scan plots to allow better visualization.

Figure 9b shows the box scan results from the {104} surface shown in Figure 8d. This scan also shows an enrichment of La near the surface and confirms the thermodynamic driving force directing La atoms to all interfaces in LCO. It appears the lanthanum has such a strong segregation potential that there is no preferential doping of specific interfaces, and it distributes across all surfaces and grain boundaries shown here. This conclusion matches the atomistic calculations of lanthanum segregation that revealed lanthanum had one of the highest segregation energies compared to the dopants studied in all four of the constructed interfaces. Noteworthy, in both segregation profiles, one observes that oxygen dips when La peaks at the interfacial regions. That suggests that La does not simply replace Co, as assumed in our atomistic calculations but that more complex reactions might be occurring. However, the observed experimental segregations confirm the trends regarding the relative segregation potential of different dopants are reasonable despite this approximation.

## Discussion

The atomistic simulations enabled screening over many dopants that could potentially segregate to surfaces and grain boundaries of LCO. The motivation was to find dopants that would potentially lower excess energies in the system, enabling greater thermodynamic stability in nanocrystalline cathodes. Out of the proposed dopants, La^3+^ had one of the highest segregation energies and therefore was selected for the experimental studies. The synthesis and characterization demonstrated La^3+^ ions segregated to surfaces and grain boundaries as predicted by the simulations. The results are very encouraging since a simulation-informed design of experiments provides a methodology for relatively quickly and inexpensively streamlining experimental investigations. The method helps overcome the existing challenges in obtaining experimental thermodynamic data on interfacial energies and segregation enthalpies in oxides and could open new opportunities in other complex oxides for batteries or other applications.

Although segregation is not a new concept in cathode doping, the connection between ion segregation and interface thermodynamic stability makes this work very relevant to the development of stable nanomaterials (and micro) for lithium-ion battery technologies, which can extend the battery’s lifetime.^58^ Additionally, to improve cyclability, the computational model helps determine the tendencies of segregation for different dopant chemistries to specific interfaces for the design of purposefully anisotropic particles. In LCO, the {001} surface is not an active surface for lithium diffusion, and the {104} surface is one of the most active surfaces since lithium ions prefer to move along layers and not across cobalt layers.^59^ This model can design specific morphology particles with an optimized fraction of {104} surfaces that in turn will enhance the lithium diffusion and battery performance. Additionally, it is reported that lithium diffusion along grain boundaries can play a critical role in the electrochemical performance of cathodes.^60,61^ The stabilization of grain boundary networks can be critical for preventing failure mechanisms like intergranular cracking or coarsening and morphological changes during electrochemical cycling.^1,62−64^ In theory, future models could be developed to design ionically and electronically conductive grain boundaries for fast lithium and electron transport. This type of energetic and morphological engineering is only possible due to the segregation behavior of dopants in nanoscale materials, and more thermodynamic understanding is necessary.

In truth, there were a number of assumptions and limitations in the atomistic simulations that enabled the extensive search through ten different dopants with varying ionic size and charge across the four structures considered. One of the most relevant approximations in the interatomic potentials was fixing the cobalt oxidation state to the trivalent state. It is well known that cobalt can assume several oxidation states in LCO, especially during lithium cycling. Hence, some changes to segregation energy values may occur if the cobalt was allowed to change oxidation state near an interface or in the presence of aliovalent dopants. However, despite multiple attempts, the study could not find interatomic potential parameters for a charge-transfer model that could accommodate the wide range of studied dopants. A charge-transfer potential that could accommodate a subset of dopants and delithiated structures could provide insight into segregation behavior as cathodes are cycled within the battery. Related to this point, particularly when aliovalent dopants are considered, other charge-compensating reactions might also occur to stabilize the incorporation of those dopants. Indeed, past work has shown such effects at grain boundaries.^65^ However, we expect that our results are still useful for identifying dopants with higher tendencies to segregate to surfaces and interfaces. Finally, only low surface energy surfaces and low-index grain boundaries were evaluated for this work. It would be valuable for future work to construct higher energy interfaces and more surfaces to look for other trends in more complex structures and as a predictive tool for morphology evolution.

## Conclusions

Atomistic simulations were used to construct four interfaces, two low energy surfaces, and two low-index grain boundaries and study the dopant segregation behavior. By inserting dopants into the bulk of the structure and at the interface, the segregation energies of ten dopants with different ionic radii and charges were calculated. The results demonstrated the linear dependence of segregation energy on the ionic radius of the dopant, where dopants with larger ionic radius had higher segregation energies. Additionally, dopants with a higher oxidation state exhibited higher segregation energy than other dopants of the same ionic size but lower oxidation state. For example, Zr^4+^ and Mg^2+^ have a similar ionic radius, but Zr^4+^ had larger segregation energies for all four interfaces studied. The magnitude of the segregation energy was highly dependent upon the specific surface and grain boundary structure. This behavior shows that the thermodynamic driving forces of each dopant depend not only on the chemical nature of the dopant but also on the detailed interfacial atomic environment.

The results were validated by experimentally synthesizing LCO nanoparticles with a dopant showing favorable segregation energy, lanthanum, and observing the segregation behavior with STEM-EELS. The hydrothermal synthesis yielded platelike nanoparticles, and the STEM-EELS images revealed clear lanthanum segregation to both surfaces, {001} and {104}, and grain boundaries. The consistency with the simulation data suggests that despite the assumptions and approximations, atomistic modeling is a viable tool for informing the experimental design and limiting the number of synthesis experiments during dopant selection for improving the performance of nanocrystalline materials.

## Supporting Information Available

Simulation special cases revealing some segregation anomalies and how they were dealt with; segregation profiles to {100} surfaces and Σ5 grain boundary; results for Al^3+^ and V^5+^ segregation to Σ3 and Σ5 grain boundaries, and to {104} surfaces (PDF)
