# Supplementary material for: Atomistic Simulation Informs Interface Engineering of Nanoscale LiCoO2
Source: Chem Mater. 2022 Aug 19;34(17):7788–98. doi: 10.1021/acs.chemmater.2c01246 (PMC9476657; doi:10.1021/acs.chemmater.2c01246)
Supplement: Supplementary file 1 — cm2c01246_si_001.pdf [file cm2c01246_si_001.pdf]

Supplemental Information

Atomistic Simulation Informs Interface Engineering of Nanoscale $LiCoO_2$

Spencer Dahl[a], Toshihiro Aoki[b], Amitava Banerjee[c], Blas Pedro Uberuaga[d], Ricardo H.R. Castro[a,*]

a Department of Materials Science and Engineering, University of California, Davis, California, 95616, USA
b Irvine Materials Research Institute (IMRI), University of California, Irvine, California, 92697, USA
c Department of Metallurgical and Materials Engineering, Indian Institute of Technology Jodhpur, Rajasthan, 342030, India
d Materials Science and Technology Division, Los Alamos National Laboratory, Los Alamos, NM 87545, USA

*Corresponding author: Ricardo H.R. Castro, rhrcastro@ucdavis.edu

Atomistic Simulation

A few unique cases from the simulations with aliovalent dopants arose during the dopant replacements. **Figure S1a** illustrates the effect of Mg^{2+} doping at different positions on the relative energy of the simulation cell for the {001} surface. During the doping study of the original structure size, it was observed that aliovalent dopants prefer to remain in the bulk structure; however, a stable or constant energy profile in the bulk region in between the surfaces was never established in this cell (in contrast to the profiles shown in Figure 2 and 3). To resolve this issue, the structure was expanded in the z direction (the surface normal direction) and in the x/y directions (the in-plane directions of the surface). The expanded z direction structures followed the same trend and never achieved a constant bulk energy, but the expanded structure in the x/y directions corrected the issue and achieved a similar segregation profile to the isovalent dopants. The hypothesis for this behavior is that the aliovalent dopants perturb the electroneutrality of the cell and in this surface structure the dopants cause strong interactions across the periodic boundary conditions in the x and y directions, leading to significant self-interaction, though why this becomes stronger as the z dimension of the structure is increased is

not clear. Further studies using a charge transfer potential could be useful for studying the effect of aliovalent dopants as the cobalt will likely help accommodate the change in electroneutrality.

Figure S1b shows aliovalent doping behavior of the Σ5 grain boundary and the impact of dopant position on the simulation cell energy. Due to some slight reconstructions at the interface, a dipole forms, causing an electric field to form across the cell that results in a small negative slope for the bulk behavior of the tetravalent dopants and a positive slope for the divalent dopants. To account for the slope, we first fit a line to the energies of the dopants in the bulk. We then used the value of this fit at the GB center as the bulk reference and measured the segregation energy of the lowest energy site relative to this value, as illustrated in **Figure S1b**. Although these effects from aliovalent dopants could have impacts on the absolute value of the segregation energies, the relative trends relating to ionic size and charge should remain consistent. The other interesting result of the Σ5 boundary doping for all oxidation states is the disorder that is seen at each grain boundary. When **Figure 3** and **Figure S1b** are compared, it can be seen that there are only a few stable sites near the boundary for the dopants in the Σ3 system while the Σ5 system shows several more local minimum positions for the dopants. This again confirms the higher structural disorder at the Σ5 interface and why the relative magnitudes of the dopant segregation energy increase with the higher index grain boundaries.

(a)

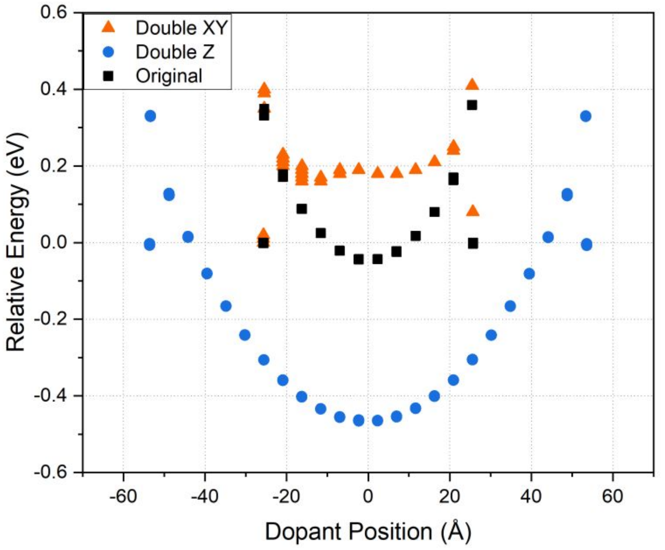

(b)

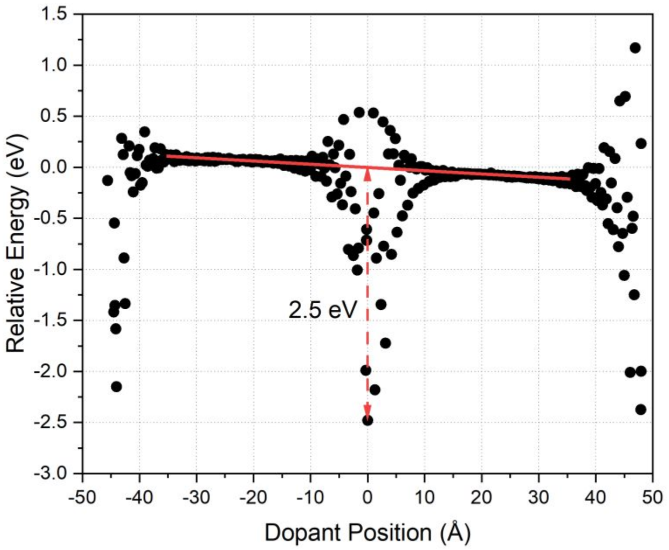

Figure S1. (a) Segregation profile of Mg^{2+} doping of {001} surface at the original structure size, structure doubled in the z direction, and structure doubled in both the x and y direction. In this plot, the surface site is located at a value of 0 eV instead of the bulk values for visualization of the data. The segregation energy can be determined from wherever the bulk energy stabilizes at the 0Å dopant position. (b) Segregation profile of Ti^{4+} doping of Σ5 grain boundary. The fitted red line shows the correction for the bulk energy value due to the dipole formation in the structure.

Table S1. Interatomic pair potential parameters for Al^{3+} and V^{5+} dopants.

Ionic Pair	A (eV)	ρ (Å)	C (eV*Å^6)
$Al^{3+} \cdots O^{2-}$ [1]	1474.4	0.3006	0
$V^{5+} \cdots O^{2-}$ [2]	2779.85	0.29185	0

(a)

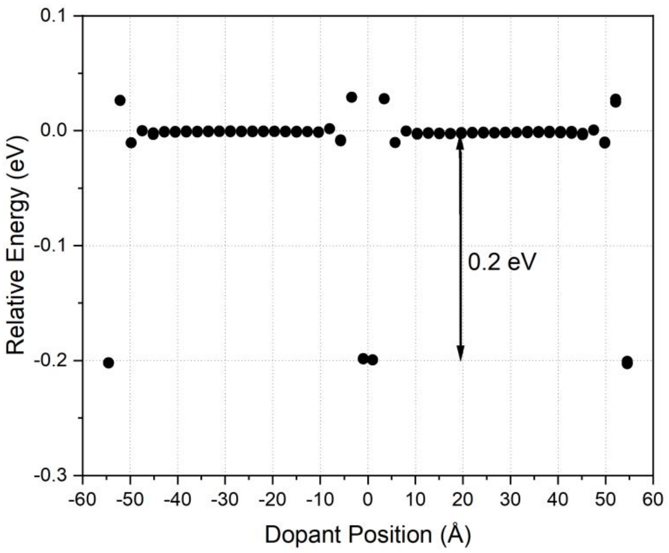

(b)

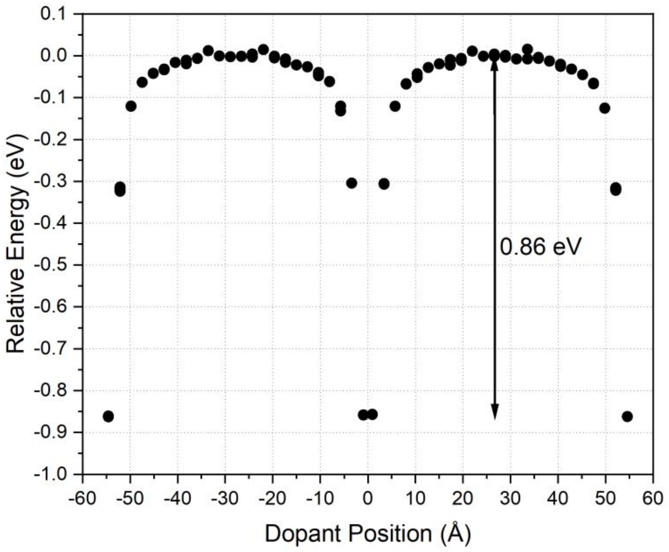

(c)

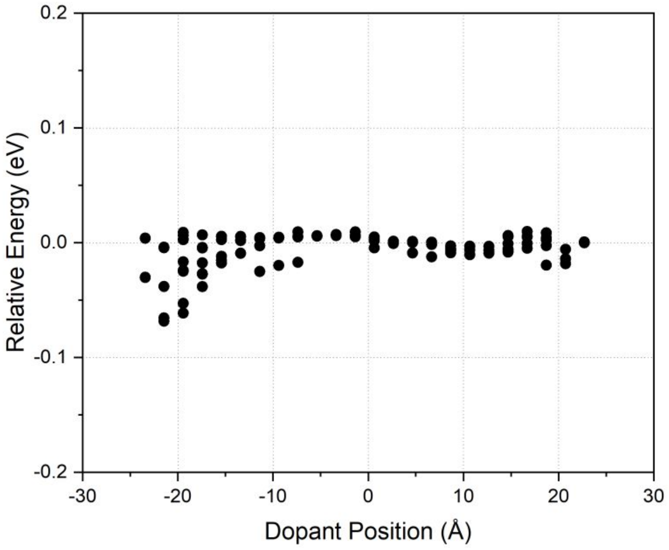

(d)

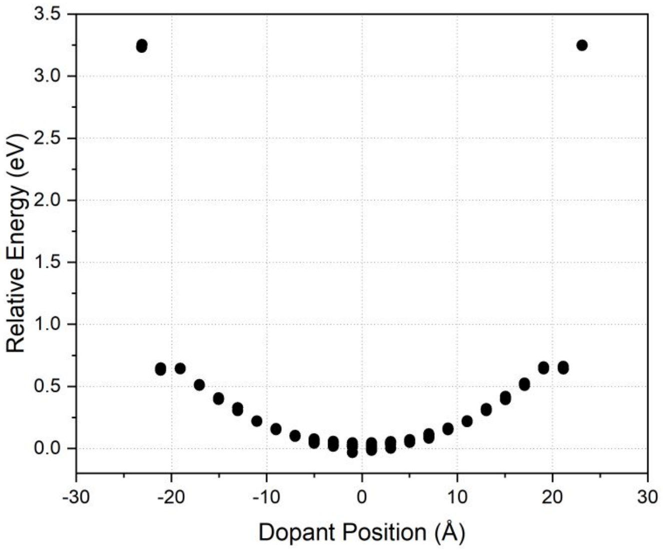

Figure S2. Segregation profile of (a) Al^{3+} to the Sigma 3 grain boundary, (b) V^{5+} to the Sigma 3 grain boundary, (c) Al^{3+} to the {104} surface, and (d) V^{5+} to the {104} surface.

(1) Sayle, D. C.; Sayle, T. X. T.; Parker, S. C.; Harding, J. H.; Catlow, C. R. A. The Stability of Defects in the Ceramic Interfaces, MgO MgO and CeO2 Al2O3. *Surf. Sci.* **1995**, *334* (1–3), 170–178. https://doi.org/10.1016/0039-6028(95)00396-7.

(2) Dietrich, A.; Catlow, C. R. A.; Maigret, B. Structural Modelling of Vanadium Pentoxide.

Mol. Simul. **1993**, *11* (5), 251–265. https://doi.org/10.1080/08927029308022513.
